# Shades of grey; Assessing the contribution of the magno‐ and parvocellular systems to neural processing of the retinal input in the human visual system from the influence of neural population size and its discharge activity on the VEP

**DOI:** 10.1002/brb3.860

**Published:** 2018-02-01

**Authors:** Valentine L. Marcar, Silvana Baselgia, Barbara Lüthi‐Eisenegger, Lutz Jäncke

**Affiliations:** ^1^ Neurorehabilitation and Paraplegic Unit REHAB Basel Basel Switzerland; ^2^ BORL Department of Neonatology University of Zürich University Hospital Zürich Zürich Switzerland; ^3^ Institute of Psychology Chair of Neuropsychology, University of Zürich Zürich Switzerland; ^4^ Praxis Barbara Lüthi‐Eisenegger Küsnacht Switzerland

**Keywords:** action potential, contrast response function, local field potential, neural mass, phasic and tonic response

## Abstract

**Introduction:**

Retinal input processing in the human visual system involves a phasic and tonic neural response. We investigated the role of the magno‐ and parvocellular systems by comparing the influence of the active neural population size and its discharge activity on the amplitude and latency of four VEP components.

**Method:**

We recorded the scalp electric potential of 20 human volunteers viewing a series of dartboard images presented as a pattern reversing and pattern on‐/offset stimulus. These patterns were designed to vary both neural population size coding the temporal‐ and spatial luminance contrast property and the discharge activity of the population involved in a systematic manner.

**Results:**

When the VEP amplitude reflected the size of the neural population coding the temporal luminance contrast property of the image, the influence of luminance contrast followed the contrast response function of the parvocellular system. When the VEP amplitude reflected the size of the neural population responding to the spatial luminance contrast property the image, the influence of luminance contrast followed the contrast response function of the magnocellular system. The latencies of the VEP components examined exhibited the same behavior across our stimulus series.

**Conclusions:**

This investigation demonstrates the complex interplay of the magno‐ and parvocellular systems on the neural response as captured by the VEP. It also demonstrates a linear relationship between stimulus property, neural response, and the VEP and reveals the importance of feedback projections in modulating the ongoing neural response. In doing so, it corroborates the conclusions of our previous study.

## 1 INTRODUCTION

“Once you eliminate the impossible, whatever remains, no matter how improbable, must be the truth”.

Arthur C. Doyle (1859–1930)

Almost a century after the introduction of electroencephalography (EEG), the quantitative relationship between stimulus property, neural response, and the electric potential measured at the scalp is still unresolved. After three decades of mapping, the anatomical make‐up of neural macro‐networks serving cognition and perception using functional magnetic resonance imaging (fMRI), understanding the interactions between and within neural macro‐network is emerging as a new challenge in neuroscience. Modulation of the neural response by these interactions occurs at a time scale of milliseconds. The high temporal resolution of EEG makes it the most cost‐effective and non‐invasive means to investigate interactions between and within neural macro‐networks. To do so, requires a quantitative understanding of the relationship between stimulus property, neural response, and the electric potential measured at the scalp.

The neural response associated with the processing of a specific event is captured by the evoked potential (EP), obtained by averaging the electric potential from repeated occurrences of the event (Monnier & Von Berger, 1953). The EP arises from a change in the ionic current flowing between apical dendrites and soma of pyramidal cells; a current that is driven by the local field potential resulting from the action of all excitatory and inhibitory post‐synaptic potentials acting at the apical dendrites of pyramidal cells (Creutzfeldt, Rosina, Ito, & Probst, 1969). The EP therefore signals a change in the neural response carried by all active neurons, rather than by the change in response of a select neural population (Celesia, 1993).

Because the anatomic and functional properties of the primate visual system are well understood, it serves as a favorite site for investigating the relationship between stimulus property, neural response, and the visual evoked potential (VEP). A linear relationship between neural discharge activity and VEP has been demonstrated (Lehmann & Skrandies, 1982). The relationship between stimulus property and the VEP is less clear; with some authors reporting it to be non‐linear (Fortune & Hood, 2003), others reporting it to be linear (Armington, 1968). Within the human visual system two distinct mechanism have been identified. These have been described as a phasic and tonic neural mechanism (Kulikowski & Tolhurst, 1973; Tolhurst, 1975) or a luminance‐ and contrast mechanism (Victor & Zemon, 1985; Zemon & Gordon, 2006). Any nonlinearity between stimulus property and the VEP must arise from a modulation of the neural response resulting from the interaction of the neural activity associated with each of these processing mechanisms. If this is the case, it should be possible to account for any nonlinearity between stimulus property and the VEP, by considering how the neural responses are associated with temporal‐ and spatial luminance contrast processing interact.

In a previous investigation, we linked the phasic neural response to temporal luminance contrast processing and the tonic neural response to spatial luminance contrast processing by considering the effect of a change in the size of the neural population on the VEP (Marcar & Jäncke, 2016). Although the association between temporal luminance processing and the magnocellular neural system and spatial luminance contrast processing and the parvocellular system is generally accepted (Derrington & Lennie, 1984), the view that magnocellular neurons respond in a more phasic manner than parvocellular neurons (Crook, Lange‐Malecki, Lee, & Valberg, 1988; Schiller, 2010) is not (Levitt, Schumer, Sherman, Spear, & Movshon, 2001).

To corroborate the link between the phasic neural response and the magnocellular system on the one hand and the tonic response and the parvocellular system on the other, we extended our initial approach of varying the size of the active neural population, by also varying the discharge activity of the active neural population. We did so by varying the luminance contrast of the stimulus elements as well as the total stimulus area occupied by these elements.

The contrast response function of the magno‐ and parvocellular systems differs so that differences in discharge activity arising from changes in luminance contrast of stimulus elements should reveal the contribution of the magno‐ and parvocellular systems. Using differences in their contrast response function to distinguish the contribution of the magno‐ and parvocellular systems has been strongly criticized (Skottun, 2014, 2015). In line with their concerns, we defined the dartboard elements using four Michelson contrasts levels. We set the lowest luminance contrast level below the saturation level of the magnocellular system, reported to lie between 16% and 32% and the higher luminance contrast levels above level at which the response of the parvocellular system increases linearly with luminance contrast (Green et al., 2009). A neural response driven by magnocellular neural activity will see the VEP amplitude at a luminance contrast level below its saturation threshold differ markedly from that observed at a luminance contrast level above this threshold. At luminance contrasts levels above the magnocellular saturation threshold, little difference in VEP amplitude should be observed. A neural response driven by parvocellular neural activity will manifest itself as a linear increase in VEP amplitude as luminance contrast level increases.

## 2 MATERIALS AND METHODS

### 2.1 Participants

In all, 20 healthy volunteers participated (10 females; mean age: 24.6 years: range 26–46 years). None had a history of neurologic illness and all reported normal vision. Participants provided their written, informed consent prior to participate in the study. The study protocol was approved by the local ethics committee (E‐08/2006, SPUK‐ Psychiatry, Zürich, Switzerland).

### 2.2 Stimulus material

A detailed description of the stimuli is provided in Marcar & Jäncke (2016). The stimulus set consisted of a disc and five dartboards. In the latter, the area covered by the white elements was 75%, 50%, 37.5%, 25%, and 12.5% of the total area of the disc. These patterns will be referred to as “Disc,” “DB75,” “DB50,” “DB37.5,” “DB25,” and “DB12.5.” At the viewing distance of 1 m, images extended from the center of gaze to an eccentricity of 8.5º.The luminance of the light elements was 145, 63, 28 cd/m^2^, or 15 cd/m^2^. The luminance of the background was 9 cd/m^2^ (Minolta: LS 110; Osaka, Japan). This resulted in a Michelson contrast of 0.90, 0.75, 0.50, or 0.25. All images were stored on the hard disk using the 8bit portable network graphic (PNG) format. This provided 256 grey levels. The lowest Michelson contrast was below the saturation threshold of the magnocellular system, whereas the higher Michelson contrasts were above this threshold (Green et al., 2009).

**Figure 1:**
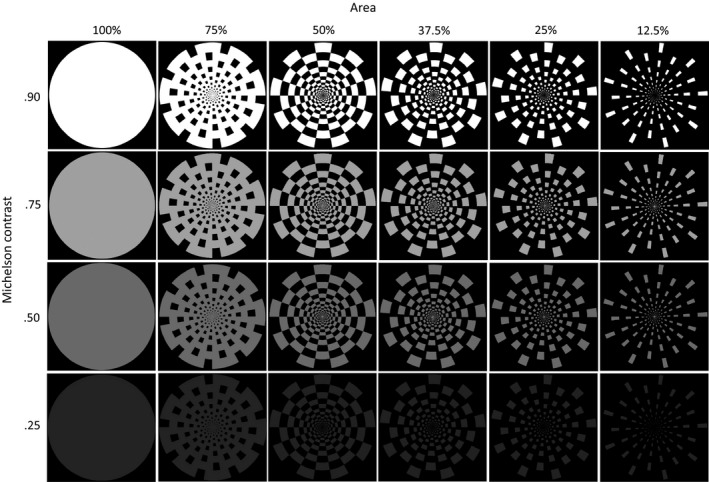
The figure depicts the disc and dartboard images used to generate the pattern reversing and pattern on‐/offset stimuli. Luminance contrast levels depicted do not correspond to the Michelson contrast actually used and serve illustration purposes only

Figure 1 shows the six patterns at different luminance contrast used to generate the pattern reversing and pattern onset/offset stimuli of our study.

### 2.3 Pattern reversing stimuli

Our four pattern reversing stimuli were generated using four complementary pairs of the DB50, DB37.5, DB25, and DB12.5 by rotating the original pattern by π radians. The characteristic of each image pair was identical so that there was no change in mean luminance following an exchange of image. All images were presented so that the center of the image coincided with the center of the monitor. Each image was presented for 500 ms (ISI) before being exchanged by its complementary image. This resulted in two reversals per second. Each pattern reversing stimulus was presented for 60 s, generating 120 reversal events.

### 2.4 Pattern onset/offset stimuli

The images used to generate our pattern on‐/offset stimuli were a DB75, DB50, and DB25 dartboard image and disc. A pattern onset/offset stimulus was generated by alternating blank image with one of the images above. During the 60 s of presentation, each image was presented for 500 ms (ISI). This resulted in 60 onset and 60 offset events.

We randomized the sequence of our stimuli between participants using the “Latin square” method.

### 2.5 Apparatus

Recordings were performed in the laboratories of the Psychology Institute of the University of Zurich with the participant seated in a Faraday cabin (CFW, Heiden, Switzerland). Participants were instructed to keep head motion and eye blinking to a minimum and to fixate the center of the image. The stimuli were presented on a 17 inch monitor (Philips 107T4, Amsterdam, The Netherlands, RRIS: SCR_008656) using a Quadro4 700XGL graphics card (NVIDIA Corporation, Santa Clara, CA, USA). The monitor brightness was set to 80%, the contrast to 95%. Each image exchange was synchronized to the vertical refresh signal of the monitor, set at 75 Hz.

### 2.6 EEG recording

The scalp electric potential was recorded using 30 Ag/Ag electrodes positioned according to the international 10/10 system (Chatrian, Lettich, & Nelson, 1985) using an electrode cap “EasyCap” (MES GMBH, Gilching, Germany). The electrode positions used were as follows: Fp1/2, F3/4, F7/8, Fz, FT7/8, FC3/4, FCz, T7/8, C3/4, Cz, TP7/8, CP3/4, CPz, P3/4, P7/8, Pz, O1/2, and Oz. Two additional electrodes were placed below the left and right zygomatic bone to record eye movements. To minimize muscle artifacts, the participants' head rested on a chin and forehead rest (Richmond Products Inc., Albuquerque, NM, USA). The EEG data were recorded and stored on a workstation using the software “Brain Vision Recorder” (Brain Products, Munich, Germany, RRID: SCR_009443). The presentation of each image was accompanied by the placing of a unique marker in the EEG‐data.

### 2.7 Post‐processing

The EEG data were processed offline using the software “Brain Vision Analyser” (Brain Products, Munich, Germany, RRID: SCR_002356). EEG data were bandpass filtered removing oscillations below 0.5 Hz and above 40 Hz and set a limit on the slope of 24 dB/oct and 48 dB/oct. Blinking and muscle artifacts in the EEG were identified by performing an independent component analysis (ICA) and removed. Any remaining artifacts were located by visual inspection of the data and marked. The signal from each electrode was re‐referenced to the average signal from all electrodes, excluding the two ocular electrodes. The start of a specific stimulus in the EEG data was located and a baseline correction performed on each epoch.

We calculated the VEP for each stimulus by averaging the 500 ms epochs starting from its identifying marker. The VEP to our pattern reversing stimuli was derived by pooling the epochs following each image exchange but separate VEP was calculated for pattern on‐ and offset. The VEP from a stimulus provided us with an assessment of the time‐locked, neural response during processing of that stimulus (Fender, Beeler, & Lehmann, 1966). We focused on the VEP from electrode Oz as it is most closely associated with the activity of striate cortex (Papakostopoulos, Hart, Corrall, & Harney, 1996; Srebro, 1987).

### 2.8 Pattern reversing

Following the ISCEV guidelines (Odom et al., 2010), we identified the N75, P100, and N135 components in the VEP to our pattern reversing stimuli. We also identified a fourth component with a positive electric potential at 240 ms. This component has been linked to perception closure (Doniger, Foxe, Murray, Higgins, & Javitt, 2002). We will refer to it as P240. For each component and subject, we determined the peak deflection amplitude. For the N75, it was the minimum between 50 and 100 ms; for the P100, the maximum between 70 and 120 ms; for the N125, the minimum between 100 and 140 ms; and for the P240, the maximum between 200 and 350 ms. The time point of the peak served as a component's latency.

### 2.9 Pattern on‐/offset

In the VEP following pattern onset and pattern offset, we identified four components at time points corresponding to the VEP components to the pattern reversing stimuli. Following the ISCEV guidelines, we will refer to them as follows: C1, P1, N1, and P2. The amplitude of C1 was the minimum in the VEP between 50 and 100 ms, that of P1 the maximum between 80 and 125 ms, that of N1 the minimum between 95 and 140 ms and that of P2 the maximum between 180 and 350 ms. The time point of the peak served as the component's latency.

### 2.10 Statistical analysis

Both amplitude and latency of the VEP components following pattern reversing, pattern on‐ and offset were compared using a multi‐factorial analysis of variance manova with repeated measures, as implemented in the General Linear Model of SPSS Ver. 22 (IBM, Armonk, NY, USA, RRID: SCR_002865). First, we compared the amplitude of all VEP components from the different presentation modes. The within‐subject factors for this comparison were as follows: MODE (Reversing, Onset, Offset). AREA (50%, 25%), CONTRAST (0.09, 0.75, 0.50, & 0.25), and COMPONENT (N75/C1, P100/P1, N135/N1, & P240/P2). We then compared the amplitude and latency of the VEP components from the pattern reversing and pattern on‐/offset stimuli separately. For the pattern reversing stimuli, the within‐subject factors were as follows: AREA (50%, 37.5%, 25%, & 12.5%), CONTRAST (0.90, 0.75, 0.50, & 0.25), and COMPONENT (N75, P100, N135, & P240). For the pattern on‐/offset stimuli, the within‐subject factors were as follows: AREA (100%, 75%, 50%, & 25%), CONTRAST (0.90, 0.75, 0.50, & 0.25), and COMPONENT (C1, P1, N1, & P2).

A featureless stimulus generates a VEP that is simpler in structure than that obtained to a patterned stimulus (Spehlmann, 1965). The disc lacks any spatial luminance contrast and can be considered a featureless stimulus and so will not elicit neural response from a mechanism selective to spatial luminance contrast. We therefore separately analyzed the influence of luminance contrast on the P1 following On‐ and Offset of the disc from that observed following on‐ and offset of the three dartboards. We restricted this analysis to P1, as this VEP component is most strongly modulated by an interaction between neural processes (Vanni et al., 2004).

Spatial frequency characteristics of the stimuli: We determined the low and high spatial frequency characteristics of our pattern using the Fourier transformation function in MatLab, Ver. 2014a (Natick, MA, USA). The low spatial frequency characteristic was represented by the power of the function F(0) and the high spatial frequency characteristic by the sum of the power in the spatial frequency range 3–7 cycles per degree (cpd). This is the range where human contrast sensitivity is highest (Campbell, Cooper, Robson, & Sachs, 1969; Leguire et al., 2011). A detailed description of the spatial frequency properties and the power in the low and high spatial frequency spectrum of our images is shown in Figure 2 of Marcar and Jäncke (2016).

## 3 RESULTS

Multiple violations of Maulchy's Sphericity were observed for most factors in the multivariate analysis of variance. We adopted the convention of Victor and Zemon and considered amplitudes of 1 μV and above to represent a genuine neural response (Victor & Zemon, 1985). Where the amplitude of a VEP component failed to reach this threshold in a specific condition, we refrained from interpreting observed differences even when statistically significant. To reduce the risk of a Type I error, we report the results from the univariate analysis of variance, with the degrees of freedom corrected following the method of Huynh‐Feldt and to maintain comparability with the findings of our previous work we rejected the NULL hypothesis if *p* ≤ .01.

First, we report the results from the analysis of the amplitudes of the VEP components. This is followed by the results of the latencies of the same VEP components. In each instance, we provide an overview by presenting the findings from the multi‐factorial analysis followed by the findings of individual factors on individual VEP components.

### 3.1 Results relating to the mode of presentation (pattern reversing vs pattern on‐/offset)

Table 1 lists the results of the multi‐factorial analysis of the VEP component amplitudes.

**Table 1:** The table contains the results from the analysis of variance of the amplitude of the VEP components to the DB50 and DB25 dartboard images presented as a pattern reversing, pattern onset and pattern offset stimulus at the four luminance contrast levels. Violations of Mauchly's Sphericity are compensated for by correcting the degree of freedom using the method of Huynh‐Feldt

Results of the analysis of variance of VEP amplitude					
Factor	*F*	*Df*	*p*	η^2^	Power
MODE	90.848	1.761	10^−3^	0.827	1.000
CONTRAST	18.427	2.483	10^−3^	0.295	0.726
AREA	7.947	1.000	.011	0.492	1.000
COMPONENT	23.352	2.957	10^−3^	0.551	1.000
MODE*CONTRAST	26.051	3.762	10^−3^	0.578	1.000
MODE*AREA	15.802	2.000	10^−3^	0.454	0.999
AREA*CONTRAST	0.671	3.000	.573	0.034	0.183
MODE*COMPONENT	31.776	4.767	10^−3^	0.626	1.000
CONTRAST*COMPONENT	18.488	4.321	10^−3^	0.493	1.000
AREA*COMPONENT	11.684	2.884	10^−3^	0.381	0.999
MODE*AREA*CONTRAST	0.261	4.420	.917	0.014	0.107
MODE*CONTRAST*COMPONENT	5.668	10.641	10^−3^	0.230	1.000
MODE*AREA*CONTRAST	17.588	5.386	10^−3^	0.481	1.000
AREA*CONTRAST*COMPONENT	2.404	8.123	.017	0.112	0.889
MODE*AREA*CONTRAST*COMPONENT	1.950	12.883	.026	0.093	0.924

The appearance of the VEP when the identical dartboard images are viewed as a pattern reversing or pattern on‐/offset differ considerably. That only two different dartboard patterns were presented in this manner, may account for why the total stimulus area undergoing a luminance contrast change failed to influence the VEP, a finding that stands in contrast to preceding work (Marcar & Jäncke, 2016). The contrast of the dartboard elements has a clear influence on the deflection amplitudes in the VEP.

### 3.2 Results involving the VEP to the pattern reversing stimuli

**Figure 2:**
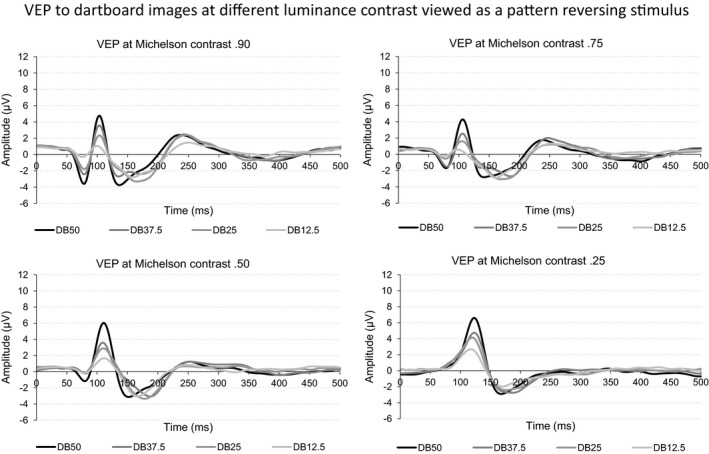
The four graphs in the figure depict the VEP obtained to the four dartboard images viewed as pattern reversing stimuli. The four graph shows the VEP obtained at Michelson contrast 0.90, 0.75, 0.50, or 0.25

The four panels of Figure 2 show the grand, mean VEP to the four dartboard images at the four Michelson contrast levels when presented as pattern reversing stimuli.

Changing the luminance contrast of the dartboard elements exerted a stronger influence on the later than the initial part of the VEP, suggesting that the subsequent processing mechanism is more sensitive to the changes in luminance contrast used in this study. This is consistent with the presence of distinct processing mechanism with different contrast response functions.

### 3.3 Results involving the amplitudes of individual VEP components to the pattern reversing stimuli

**Figure 3:**
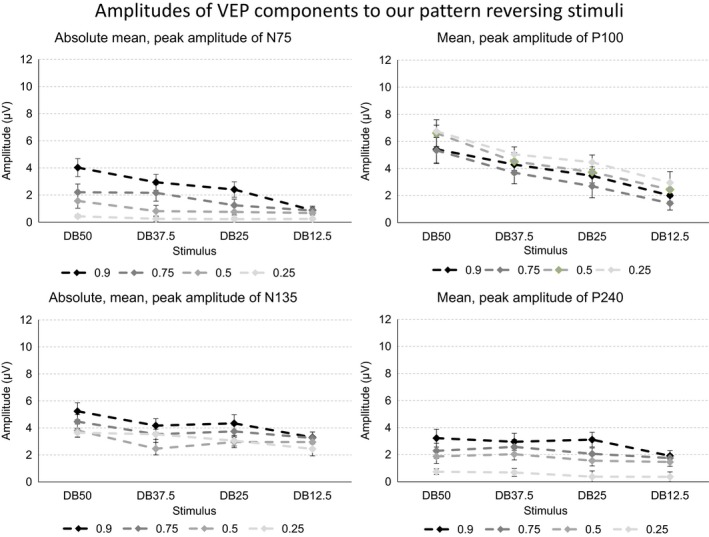
The four graphs in the figure depict the mean, peak amplitude of the four VEP components when subjects viewed the four dartboard images as a pattern reversing stimulus. The four graphs show the values obtained at Michelson contrast 0.90, 0.75, 0.50, or 0.25. The error bars indicate the standard error of the mean (SEM)

The four panels of Figure 3 depict the mean, peak amplitude of the four VEP components to the pattern reversing stimuli presented at the four Michelson contrast levels.

Increasing both the total stimulus area undergoing a change in luminance contrast and the Michelson contrast of the dartboard elements increases the amplitude of the N75 VEP component. The amplitude of the P100 and N135 VEP components, however, only reflects the change in total stimulus area undergoing a change in luminance contrast. The amplitude of the P240 VEP component reflected the Michelson contrast of the dartboard elements only.

Table 2 lists the results of the analysis of variance of the VEP component amplitudes to the pattern reversing stimuli at the four Michelson contrast levels.

**Table 2:** The table contains the results from the analysis of variance of the component amplitudes to the dartboard images presented as a pattern reversing stimulus at the four luminance contrast levels. Violations of Mauchly's Sphericity are compensated by correcting the degree of freedom using the method of Huynh‐Feldt

Analysis of variance of amplitudes to pattern reversing stimuli						
Within‐subject effect	*F*		*df*	*p*	η^2^	Power
CONTRAST	9.708		2.451	10^−3^	0.338	0.989
AREA	33.813		1.645	10^−3^	0.640	1.000
COMPONENT	12.514		2.308	10^−3^	0.397	0.997
CONTRAST*AREA	3.558		4.628	.007	0.158	0.888
CONTRAST*COMPONENT	8.345		5.080	10^−3^	0.305	1.000
AREA* COMPONENT	16.411		3.769	10^−3^	0.463	1.000
AREA*CONTRAST*COMPONENT	2.390		16.301	.002	0.112	0.990

Within‐subject contrast	Trend	*F*	*df*	*p*	η^2^	Power
CONTRAST	Linear	14.991	1	.001	0.441	0.956
AREA	Linear	41.858	1	10^−3^	0.688	1.000
	Cubic	12.402	1	.002	0.395	0.916

Within‐subject effect: N75	*F*		*df*	*p*	η^2^	Power
CONTRAST	10.926		2.489	10^−3^	0.365	0.995
AREA	16.402		1.542	10^−3^	0.463	0.996
AREA*CONTRAST	4.746		4.540	.001	0.200	0.960

Within‐subject contrast	Trend	*F*	*df*	*p*	η^2^	Power
CONTRAST	Linear	19.103	1	10^−3^	0.501	0.985
AREA	Linear	20.826	1	10^−3^	0.523	0.911

Within‐subject effect: P100	*F*		*df*	*p*	η^2^	Power
CONTRAST	5.613		2.119	.006	0.228	0.613
AREA	49.370		1.650	10^−3^	0.772	1.000
AREA*CONTRAST	1.587		9.000	.122	0.077	0.729

Within‐subject contrast	Trend	*F*	*df*	*p*	η^2^	Power
CONTRAST	Quadratic	9.338	1	.007	0.330	0.826
AREA	Linear	64.134	1	10^−3^	0.771	1.000

Within‐subject effect: N135	*F*		*df*	*p*	η^2^	Power
CONTRAST	5.236		1.924	.011	0.216	0.789
AREA	6.177		1.710	.007	0.245	0.822
AREA*CONTRAST	2.045		5.874	.067	0.097	0.714

Within‐subject contrast	Trend	*F*	*df*	*p*	η^2^	Power
AREA	Cubic	8.761	1	.008	0.316	0.802

Within‐subject effect: P240	*F*		*df*	*p*	η^2^	Power
CONTRAST	16.739		1.579	10^−3^	0.486	1.000
AREA	5.524		1.643	.013	0.225	0.763
AREA*CONTRAST	2.430		5.961	.030	0.113	0.804

Within‐subject contrast	Trend	*F*	*df*	*p*	η^2^	Power
CONTRAST	Linear	20.599	1	10^−3^	0.520	0.990

### 3.4 Results involving the VEP following pattern onset

**Figure 4:**
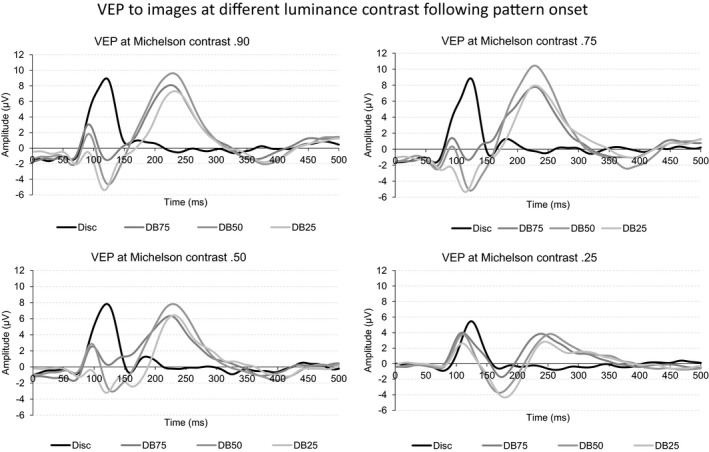
The four graphs in the figure depict the VEP following onset of the disc and the four dartboard images. The four graphs show the VEPs obtained at Michelson contrast 0.90, 0.75, 0.50, or 0.25

The four panels of Figure 4 depict the grand, mean VEP following onset of the disc, and the three dartboard images when presented at different Michelson contrast levels.

The amplitude of the deflections in the later part of the VEP is noticeably larger than during the pattern reversing stimuli. The VEP following onset of the disc is simpler in appearance than that generated by the dartboards. This simplicity arises from the lack of deflections during the latter part of the VEP. It indicates that the disc elicits no or a much weaker neural response in the neural mechanism processing spatial luminance contrast than the dartboards. We, therefore, report findings on the influence of Michelson contrast on the amplitude of individual VEP components from the disc and dartboard images separately.

### 3.5 Results involving the amplitude of individual VEP components following pattern onset

**Figure 5:**
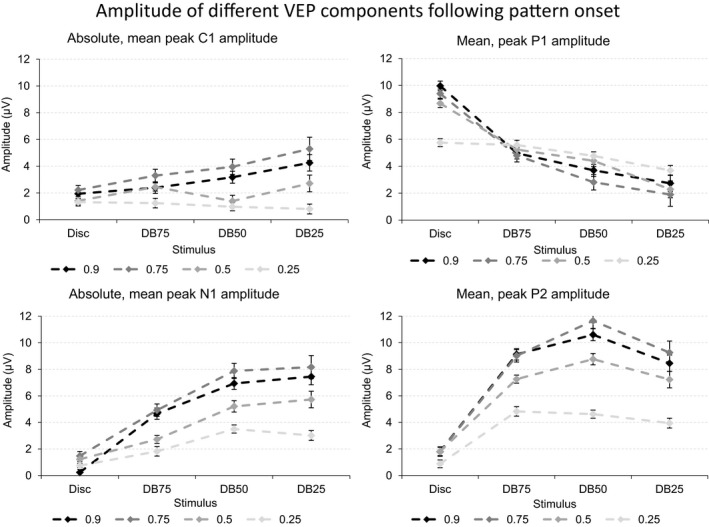
The four graphs in the figure depict the mean, peak amplitude of the four VEP components following onset of the disc, and the three dartboard images. Each graph depicts the values obtained at one of the four Michelson contrast levels 0.90, 0.75, 0.50, or 0.25. The error bars indicate the standard error of the mean (SEM)

The four panels of Figure 5 depict the mean, peak amplitude of the four VEP components following onset of the disc, and the three dartboard images when presented at the four Michelson contrast levels.

Table 3 lists the results of the analysis of variance of the VEP component amplitude following onset of the disc at the four Michelson contrasts.

**Table 3:** The table contains the results from the analysis of variance of the VEP component amplitudes following onset of the disc at the four luminance contrast levels. Violations of Mauchly's Sphericity are compensated for by correcting the degree of freedom using the method of Huynh‐Feldt

Analysis of variance of individual component amplitude						
C1						
Factor	*F*	*df*		*p*	η^2^	Power
CONTRAST	2.811	2.597		.030	0.153	0.693

Within‐subject contrast	Trend	*F*	*df*	*p*	η^2^	Power
CONTRAST	Linear	8.286	1	.010	0.304	0.780

P1						
Factor	*F*	*df*		*p*	η^2^	Power
CONTRAST	22.825	2.458		10^−3^	0.808	1.000

Within‐subject contrast	Trend	*F*	*df*	*p*	η^2^	Power
CONTRAST	Linear	63.129	1	10^−3^	0.546	1.000
	Quadratic	15.079	1	.001	0.442	0.957

N1						
Factor	*F*	*df*		*p*	η^2^	Power
CONTRAST	3.688	2.802		.019	0.163	0.446

P2						
Factor	*F*	*df*		*p*	η^2^	Power
CONTRAST	3.413	3.000		.003	0.216	0.911

Within‐subject contrast	Trend	*F*	*df*	*p*	η^2^	Power
CONTRAST	Linear	10.212	1	.005	0.350	0.858

Michelson contrast has no influence on C1 amplitude. P1 amplitude at the Michelson contrast above the saturation threshold of the magnocellular system is considerably larger than below this threshold. Michelson contrast has no influence on N1 amplitude. P2 amplitude increases linearly with Michelson contrast, though at a smaller scale than P1.

Table 4 lists the results of the multi‐factorial analysis of the VEP following onset of the dartboard images at the four Michelson contrasts.

**Table 4:** The table contains the results from the analysis of variance of the VEP component amplitudes following onset of the three dartboard images at the four luminance contrast levels. Violations of Mauchly's Sphericity are compensated for by correcting the degree of freedom using the method of Huynh‐Feldt

Analysis of variance of individual VEP component amplitude						
C1						
Factor	*F*	*df*		*p*	η^2^	Power
CONTRAST	17.635	2.338		10^−3^	0.481	1.000
AREA	6.225	1.345		.013	0.248	0.754
CONTRAST*AREA	3.779	4.129		.007	0.166	0.880

Within‐subject contrast	Trend	*F*	*df*	*p*	η^2^	Power
CONTRAST	Linear	29.251	1	10^−3^	0.606	0.999
	Quadratic	31.698	1	10^−3^	0.625	1.000
CONTRAST*AREA	Linear	15.567	1	.001	0.450	0.678

P1						
Factor	*F*	*df*		*p*	η^2^	Power
CONTRAST	2.902	2.426		.055	0.132	0.593
AREA	15.463	1.873		10^−3^	0.449	0.998
CONTRAST*AREA	0.667	5.750		.660	0.035	0.255

Within‐subject contrast	Trend	*F*	*df*	*p*	η^2^	Power
AREA	Linear	21.962	1	10^−3^	0.536	0.993

N1						
Factor	*F*	*df*		*p*	η^2^	Power
CONTRAST	27.678	1.690		10^−3^	0.593	1.000
AREA	18.403	1.938		10^−3^	0.492	1.000
CONTRAST*AREA	1.918	5.141		.096	0.092	0.636

Within‐subject contrast	Trend	*F*	*df*	*p*	η^2^	Power
CONTRAST	Linear	30.219	1	10^−3^	0.614	0.999
	Quadratic	32.031	1	10^−3^	0.628	1.000
	Cubic	11.146	1	.003	0.370	0.886
AREA	Linear	45.805	1	10^−3^	0.707	1.000

P2						
Factor	*F*	*df*		*p*	η^2^	Power
CONTRAST	32.370	1.761		10^−3^	0.630	1.000
AREA	19.871	2.000		10^−3^	0.511	1.000
CONTRAST*AREA	4.0071	5.550		.001	0.176	0.959

Within‐subject contrast	Trend	*F*	*df*	*p*	η^2^	Power
CONTRAST	Linear	38.879	1	10^−3^	0.672	1.000
	Quadratic	37.876	1	10^−3^	0.666	1.000
AREA	Quadratic	30.594	1	10^−3^	0.617	0.999

C1 amplitude does not react to the total stimulus area undergoing a luminance contrast change but increases nonlinearly with the Michelson contrast of the dartboard elements. P1 amplitude increased with total stimulus area undergoing a luminance contrast change but is impervious to the Michelson contrast of the dartboard elements. N1 amplitude increased linearly with total stimulus area undergoing a luminance contrast change but reacted in a nonlinear manner to an increase in Michelson contrast of the dartboard elements. P2 amplitude reacted in a nonlinear manner to an increase in total stimulus area undergoing a luminance contrast change as well as to an increase in Michelson contrast of the dartboard elements.

### 3.6 Results involving the VEP following pattern offset

**Figure 6:**
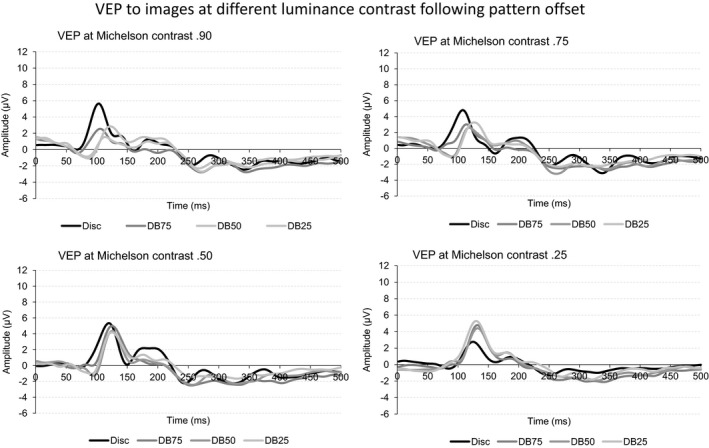
The four graphs in the figure depict the VEP obtained following offset of the four dartboard images. Each graph shows the VEP obtained at a Michelson contrast level of 0.90, 0.75, 0.50, or 0.25

The four panels of Figure 6 depict the grand, mean VEP following offset of the disc, and the three dartboard images when presented at the four Michelson contrasts.

The VEP generated following offset of our various pattern is both smaller in overall amplitude and simpler in appearance than that observed following onset of these pattern. This simplicity in appearance is attributable to the weak or absent deflections in the latter part of the VEP. While the VEP following offset of the disc decreased in amplitude with decreasing Michelson contrast the VEP following offset of the dartboards increased.

### 3.7 Results involving the amplitude of individual VEP components following pattern offset

**Figure 7:**
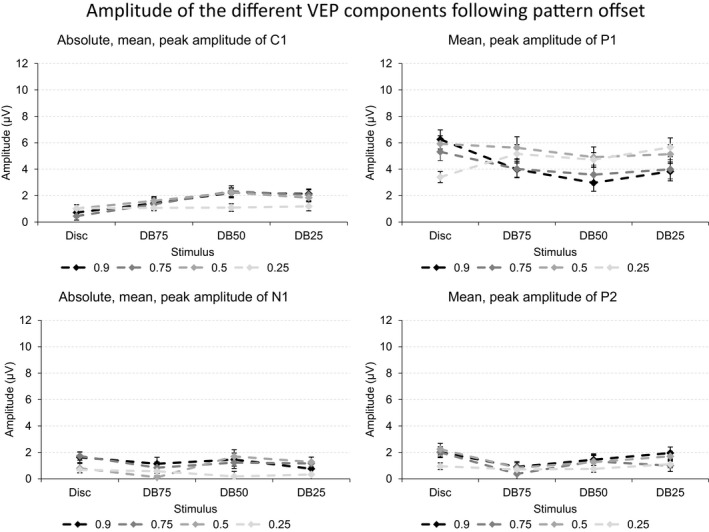
The four graphs in the figure depict the mean, peak amplitude of the four VEP components following offset of the disc, and the three dartboard images. Each graph depicts the values obtained at one of the four Michelson contrast levels 0.90, 0.75, 0.50, or 0.25. The error bars indicate the standard error of the mean (SEM)

The four panels of Figure 7 depict the mean, peak VEP amplitude following offset of the disc, and the three dartboard images when presented at four Michelson contrasts.

Table 5 lists the results of the multi‐factorial analysis involving the VEP amplitude following offset of the disc at the four Michelson contrast levels.

**Table 5:** The table contains the results from the analysis of variance of the individual VEP component amplitudes following offset of the disc at the four luminance contrast levels. Violations of Mauchly's Sphericity are compensated for by correcting the degree of freedom using the method of Huynh‐Feldt

Univariate analysis of variance of component amplitude						
C1						
Factor	*F*	*df*		*p*	η^2^	Power
CONTRAST	1.070	3.000		0.369	0.053	0.275

P1						
Factor	*F*	*df*		*p*	η^2^	Power
CONTRAST	12.652	2.678		10^−3^	0.400	0.999

Within‐subject contrast	Trend	*F*	*df*	*p*	η^2^	Power
CONTRAST	Linear	20.190	1	10^−3^	0.515	0.989
	Quadratic	12.411	1	.001	0.486	0.972

N1						
Factor	*F*	*df*		*p*	η^2^	Power
CONTRAST	4.473	3.000		.006	0.195	0.869

Within‐subject contrast	Trend	*F*	*df*	*p*	η^2^	Power
CONTRAST	Linear	8.208	1	.010	0.302	0.776

P2						
Factor	*F*	*df*		*p*	η^2^	Power
CONTRAST	4.863	3.000		.004	0.204	0.887

Michelson contrast of the disc has no influence on C1 amplitude. P1 amplitude increases nonlinearly with Michelson contrast. Its amplitude is considerably lower at the Michelson contrast below the saturation threshold of the magnocellular system than above this threshold. The N1 and P1 amplitudes also change significantly with the Michelson contrast of the disc. The amplitude of the latter reaches a lower amplitude at the Michelson contrast below the saturation threshold of the magnocellular system than above this threshold.

Table 6 lists the results of the multi‐factorial analysis involving the VEP amplitude following offset of the three dartboard images at the four Michelson contrast levels.

**Table 6:** The table contains the results from the analysis of variance of the individual VEP component amplitudes following offset of the three dartboard images at the four luminance contrast levels. Violations of Mauchly's Sphericity are compensated for by correcting the degree of freedom using the method of Huynh‐Feldt

Analysis of variance of individual VEP component amplitude						
C1						
Factor	*F*	*df*		*p*	η^2^	Power
CONTRAST	2.734	2.773		.057	0.126	0.607
AREA	4.722	2.000		.015	0.199	0.757
CONTRAST*AREA	0.494	2.466		.789	0.025	0.182

Within‐subject contrast	Trend	*F*	*df*	*p*	η^2^	Power
AREA	Quadratic	8.642	1	.008	0.313	0.796

P1						
Factor	*F*	*df*		*p*	η^2^	Power
CONTRAST	9.107	2.251		10^−3^	0.324	0.997
AREA	4.148	2.000		.023	0.179	0.698
CONTRAST*AREA	0.703	4.370		.604	0.036	0.228

Within‐subject contrast	Trend	*F*	*df*	*p*	η^2^	Power
CONTRAST	Linear	24.679	1	10^−3^	0.565	0.997
AREA	Quadratic	8.875	1	0.008	0.318	0.807

N1						
Factor	*F*	*df*		*p*	η^2^	Power
CONTRAST	2.248	2.782		.098	0.106	0.518
AREA	1.690	1.957		.199	0.082	0.330
CONTRAST*AREA	0.845	4.796		.518	0.043	0.284

P2						
Factor	*F*	*df*		*p*	η^2^	Power
CONTRAST	3.889	2.960		.014	0.170	0.795
AREA	2.718	1.886		.082	0.125	0.489
CONTRAST*AREA	0.950	6.000		.462	0.048	0.363

Undirected analysis of C1 amplitude appears unaffected by either the total stimulus area undergoing a change in luminance contrast or by the Michelson contrast of the dartboard elements. A directed analysis of the former, however, yields a nonlinear relationship between stimulus area undergoing a luminance contrast change and C1 amplitude. P1 amplitude decreases linearly with increasing Michelson contrast. Its amplitude also decreases as the total stimulus area undergoing the change in luminance contrast increases. This latter response is nonlinear, with the pattern with the largest high spatial frequency power, DB50 dartboard, yielding the lowest amplitude. N1 and P2 amplitude did not respond to either total stimulus area undergoing a luminance contrast change or to the Michelson contrast of the dartboard elements.

### 3.8 Results from the multi‐factorial analysis of the latencies of VEP components

Table 7 lists the results of the multi‐factorial analysis of variance involving all factors.

**Table 7:** The table shows the results from the analysis of variance using the General Linear Model on component latencies to the DB50 and DB25 dartboard images presented as a pattern reversing, pattern onset, and pattern offset stimulus at the four luminance contrast levels. Violations of Mauchly's Sphericity are compensated for by correcting the degree of freedom using the method of Huynh‐Feldt

Factor	*F*	*df*	*p*	η^2^	Power
MODE	17.210	1.565	10^−3^	0.475	0.997
AREA	2.439	1.000	.135	0.114	0.317
CONTRAST	14.045	2.778	10^−3^	0.425	1.000
COMPONENT	1664.921	2.001	10^−3^	0.989	1.000
MODE*AREA	1.723	1.822	.195	0.083	0.322
MODE*CONTRAST	8.296	4.901	10^−3^	0.304	1.000
MODE*COMPONENT	20.853	4.413	10^−3^	0.523	1.000
CONTRAST*AREA	2.413	2.867	.079	0.113	0.559
CONTRAST*COMPONENT	8.904	5.589	10^−3^	0.148	0.996
AREA*COMPONENT	2.924	2.496	.052	0.133	0.605
MODE*AREA*CONTRAST	0.442	4.375	.794	0.023	0.154
MODE*CONTRAST*COMPONENT	3.302	12.087	10^−3^	0.148	0.996
MODE*AREA*COMPONENT	1.461	3.859	.224	0.071	0.424
AREA*CONTRAST*COMPONENT	1.833	6.249	.095	0.088	0.679
MODE*AREA*CONTRAST*COMPONENT	0.427	11.433	.947	0.022	0.235

Of the factors MODE, AREA, and CONTRAST, only the factor AREA did not exert an influence on the latency of the VEP components. The presence of significant two‐way interactions MODE*COMPONENT and CONTRAST*COMPONENT indicates that the mode of presentation and the Michelson contrast of the dartboard elements influenced the latency of the VEP components differently.

### 3.9 Results involving latencies of VEP components to pattern reversing stimuli

**Figure 8:**
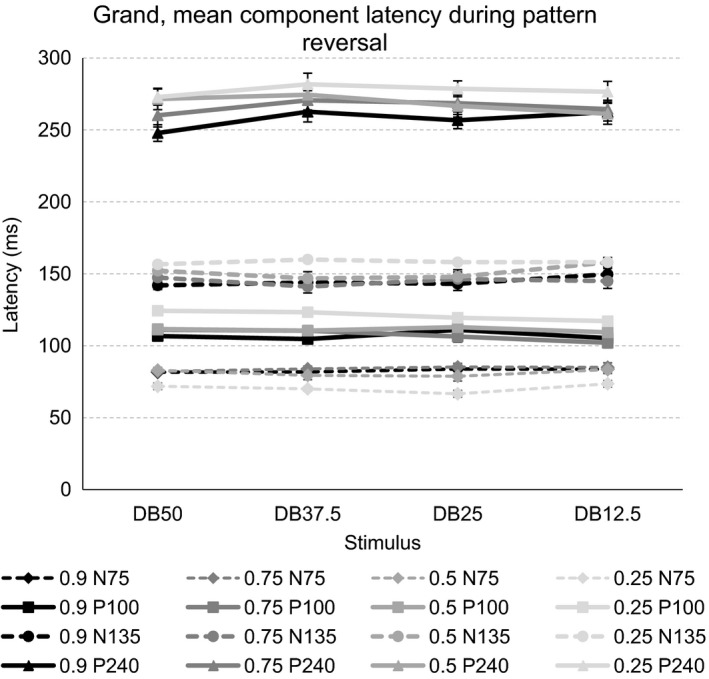
The graph shows the grand, mean latencies of the four VEP components when the four dartboard images were views as a pattern reversing stimuli at the four Michelson contrast levels. The error bars indicate the standard error of the mean (SEM)

Figure 8 shows the grand, mean latency of the VEP components to the four dartboard images when viewed as pattern reversing stimuli at four Michelson contrast levels.

Table 8 lists the results of the analysis of variance of VEP component latency to the pattern reversing stimuli.

**Table 8:** The table contains the results from the analysis of variance of the VEP component latencies when participants viewed the dartboard images presented as pattern reversal stimuli at the four luminance contrast levels. Violations of Mauchly's Sphericity are compensated for by correcting the degree of freedom using the method of Huynh‐Feldt

Analysis of variance of latencies during pattern reversing stimuli						
Within‐subject effect	*F*		*df*	*p*	η^2^	Power
CONTRAST	8.878		2.429	10^−3^	0.318	0.993
AREA	0.496		2.568	.658	0.025	0.137
COMPONENT	1337.476		2.432	10^−3^	0.986	1.000
CONTRAST*AREA	1.353		8.421	.218	0.066	0.620
CONTRAST*COMPONENT	7.466		4.000	10^−3^	0.282	0.995
AREA* COMPONENT	2.203		3.838	.080	0.104	0.608
AREA*CONTRAST*COMPONENT	0.884		13.118	.571	0.044	0.540

Within‐subject contrast	Trend	*F*	*df*	*p*	η^2^	Power
CONTRAST	Linear	21.732	1	10^−3^	0.534	0.903

Within‐subject effect: N75	*F*		*df*	*p*	η^2^	Power
CONTRAST	18.317		2.234	10^−3^	0.491	1.000
AREA	1.321		1.936	.279	0.065	0.264
AREA*CONTRAST	2.342		6.567	.031	0.110	0.818

Within‐subject contrast	Trend	*F*	*df*	*p*	η^2^	Power
CONTRAST	Linear	25.327	1	10^−3^	0.571	0.997
	Quadratic	24.548	1	10^−3^	0.564	0.997

Within‐subject effect: P100	*F*		*df*	*p*	η^2^	Power
CONTRAST	20.825		2.720	10^−3^	0.523	1.000
AREA	1.825		1.592	.184	0.088	0.316
AREA*CONTRAST	1.725		5.513	.128	0.083	0.605

Within‐subject contrast	Trend	*F*	*df*	*p*	η^2^	Power
CONTRAST	Linear	42.564	1	10^−3^	0.691	1.000
	Quadratic	13.897	1	.001	0.422	0.942

Within‐subject effect: N135	*F*		*df*	*p*	η^2^	Power
CONTRAST	10.246		2.322	10^−3^	0.350	0.990
AREA	2.246		2.503	.105	0.106	0.487
AREA*CONTRAST	2.087		5.274	.070	0.099	0.688

Within‐subject contrast	Trend	*F*	*df*	*p*	η^2^	Power
CONTRAST	Linear	17.941	1	10^−3^	0.486	0.980

Within‐subject effect: P240	*F*		*df*	*p*	η^2^	Power
CONTRAST	4.715		2.540	.008	0.199	0.830
AREA	1.760		2.325	.179	0.085	0.376
AREA*CONTRAST	0.638		8.867	.761	0.003	0.306

Within‐subject contrast	Trend	*F*	*df*	*p*	η^2^	Power
CONTRAST	Linear	10.193	1	.005	0.349	0.857

Increasing the total stimulus area undergoing a luminance contrast change has no influence the latency of the VEP components. Increasing the Michelson contrast of the dartboard elements increases VEP component latency. At the Michelson contrast below the saturation threshold of the magnocellular system, N75 latency differs markedly from its latency to Michelson contrasts above this threshold. P100 latency decreases as Michelson contrast increases. At the Michelson contrast below the saturation threshold of the magnocellular system, N135 latency is markedly longer than at the Michelson contrasts above this threshold. The latency of N135 reacts to Michelson contrast in the manner identical to the P100. The influence of Michelson contrast of the dartboard elements on P240 latency is linked to the high spatial frequency content of the pattern. With the DB50, the dartboard with the greatest high spatial frequency content, it decreases linearly with increasing Michelson contrast. With the DB12.5, the dartboard pattern with the lowest high spatial frequency content, its latency is considerably longer at the Michelson contrast below the saturation level of the magnocellular system than above this threshold level.

### 3.10 Results involving latencies of VEP components following pattern onset

**Figure 9:**
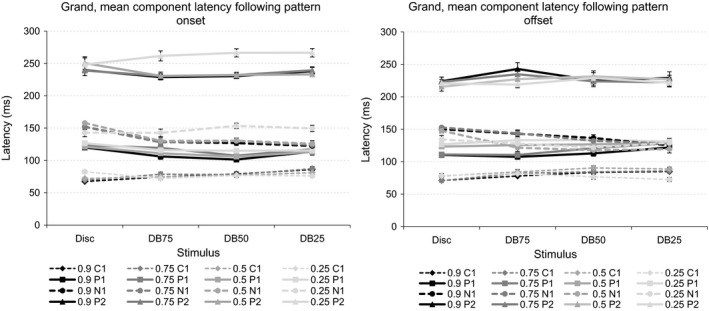
The left‐hand graph shows the grand, mean latencies of the four VEP components following onset of the disc, and the three dartboard images at the four Michelson contrast levels. The right‐hand graph shows the grand, mean latencies of the four VEP components following offset of the same images viewed at the four Michelson contrast levels The error bars indicate the standard error of the mean (SEM)

The left panel of Figure 9 depicts the grand, mean latencies of the four VEP components obtained following onset of the disc, and the three dartboard images at the four Michelson contrast levels.

Both total stimulus area undergoing a luminance contrast change as well as the Michelson contrast influenced the latency of the VEP components. The significant two‐way interaction AREA*CONTRAST indicates that the two factors influence latencies differently. The presence of the significant two‐way interactions AREA*COMPONENT and CONTRAST*COMPONENT indicates that the latency of different VEP components is influenced differently by these factors.

Table 9 lists the results of the analysis of variance of VEP component latency following pattern onset at the four Michelson contrast levels.

**Table 9:** The table contains the results from the analysis of variance of the latencies of the VEP components following the onset of the disc and dartboards presented at the four luminance contrast levels. Violations of Mauchly's Sphericity are compensated for by correcting the degree of freedom using the method of Huynh‐Feldt

Analysis of variance of latencies following pattern onset						
Within‐subject effect	*F*		*df*	*p*	η^2^	Power
CONTRAST	23.657		2.280	10^−3^	0.555	1.000
AREA	12.712		2.146	10^−3^	0.401	0.996
COMPONENT	826.743		2.184	10^−3^	0.978	1.000
CONTRAST*AREA	4.612		8.827	10^−3^	0.195	0.998
CONTRAST*COMPONENT	5.153		3.604	.002	0.213	0.972
AREA* COMPONENT	5.249		4.364	.001	0.216	0.972
AREA*CONTRAST*COMPONENT	3.325		13.514	10^−3^	0.149	0.998

Within‐subject contrast	Trend	*F*	*df*	*p*	η^2^	Power
CONTRAST	Linear	37.695	1	10^−3^	0.665	1.000
	Quadratic	12.504	1	.002	0.397	0.918
	Cubic	10.841	1	.004	0.363	0.877
AREA	Linear	6.752	1	.018	0.262	0.693
	Quadratic	23.080	1	10^−3^	0.548	0.995

Within‐subject effect: C1	*F*		*df*	*p*	η^2^	Power
CONTRAST	1.219		2.977	.311	0.060	0.308
AREA	8.758		2.203	10^−3^	0.316	0.970
AREA*CONTRAST	5.271		9.000	10^−3^	0.217	1.000

Within‐subject contrast	Trend	*F*	*df*	*p*	η^2^	Power
AREA	Linear	14.810	1	.001	0.427	0.946

Within‐subject effect: P1	*F*		*df*	*p*	η^2^	Power
CONTRAST	2.893		2.483	.054	0.132	0.599
AREA	11.150		2.501	10^−3^	0.370	0.996
AREA*CONTRAST	1.024		7.459	.419	0.051	0.445

Within‐subject‐contrast	Trend	*F*	*df*	*p*	η^2^	Power
AREA	Linear	8.840	1	.008	0.318	0.805
	Quadratic	24.463	1	10^−3^	0.563	0.997

Within‐subject effect: N1	*F*		*df*	*p*	η^2^	Power
CONTRAST	8.512		1.507	10^−3^	0.309	0.991
AREA	18.847		1.912	10^−3^	0.489	1.000
AREA*CONTRAST	8.172		5.231	10^−3^	0.301	1.000

Within‐subject contrast	Trend	*F*	*df*	*p*	η^2^	Power
CONTRAST	Linear	11.130	1	.003	0.369	0.886
AREA	Linear	27.258	1	10^−3^	0.595	0.999
	Quadratic	9.517	1	.006	0.334	0.833
	Cubic	15.505	1	.001	0.449	0.962

Within‐subject effect: P2	*F*		*df*	*p*	η^2^	Power
CONTRAST	11.494		1.948	10^−3^	0.377	0.988
AREA	0.824		1.899	.441	0.042	0.177
AREA*CONTRAST	2.377		5.484	.039	0.111	0.767

Within‐subject contrast	Trend	*F*	*df*	*p*	η^2^	Power
CONTRAST	Linear	14.055	1	.001	0.426	0.944
	Quadratic	14.905	1	.001	0.440	0.955

C1 latency is unaffected by Michelson contrast but increases as the total stimulus area undergoing a luminance contrast change decreases. Its latency following onset of the disc at the Michelson contrast below the saturation threshold of the magnocellular system is considerably longer than at the Michelson contrasts above this threshold. The highly significant two‐way interaction AREA*CONTRAST implies that these two factors modulated its latency differently. P1 latency does not vary with Michelson contrast but does vary with total stimulus area undergoing a luminance contrast change. N1 latency increases as the total stimulus area undergoing a change in luminance contrast increases but decreases as Michelson contrast increases. At the Michelson contrast below saturation threshold of the magnocellular system, its latency is considerably longer than above this threshold. The highly significant two‐way interaction AREA*CONTRAST shows that these two factors influenced the latency of this VEP component differently. P2 latency is unaffected by the total stimulus area undergoing a luminance contrast change but is strongly influenced by Michelson contrast. At the Michelson contrast below the saturation level of the magnocellular system, its latency is considerably longer than above this threshold. There is also little difference in its latency between the Michelson contrasts above the saturation threshold of the magnocellular system.

### 3.11 Results involving latencies of VEP components following pattern offset

The right panel of Figure 9 depicts the grand, mean latencies of the four VEP components obtained following offset of the disc, and the three dartboard images at the four Michelson contrast levels.

Table 10 lists the results of the analysis of variance of VEP component latency following pattern offset at the four Michelson contrast levels.

**Table 10:** The table shows the results from the analysis of variance of the component latencies following the offset of the disc and dartboards presented at the four luminance contrast levels. Violations of Mauchly's Sphericity are compensated for by correcting the degree of freedom using the method of Huynh‐Feldt

Analysis of variance of latencies following pattern offset						
Within‐subject effect	*F*		*df*	*p*	η^2^	Power
CONTRAST	0.329		2.840	.804	0.017	0.108
AREA	0.152		2.607	.907	0.008	0.075
COMPONENT	766.119		2.674	10^−3^	0.976	1.000
CONTRAST*AREA	0.269		6.204	.954	0.014	0.122
CONTRAST*COMPONENT	5.542		6.818	10^−3^	0.226	1.000
AREA* COMPONENT	7.981		5.781	10^−3^	0.296	1.000
AREA*CONTRAST*COMPONENT	1.706		16.298	.043	0.082	0.931

Within‐subject effect: C1	*F*		*df*	*p*	η^2^	Power
CONTRAST	7.151		2.382	.001	0.273	0.945
AREA	2.024		2.429	.135	0.096	0.437
AREA*CONTRAST	3.116		6.486	.006	0.141	0.922

Within‐subject contrast	Trend	*F*	*df*	*p*	η^2^	Power
CONTRAST	Quadratic	17.145	1	.001	0.474	0.975

Within‐subject effect: P1	*F*		*df*	*p*	η^2^	Power
CONTRAST	22.103		2.613	10^−3^	0.538	1.000
AREA	13.696		2.947	10^−3^	0.419	1.000
AREA*CONTRAST	2.540		6.422	.021	0.118	0.846

Within‐subject contrast	Trend	*F*	*df*	*p*	η^2^	Power
CONTRAST	Linear	46.127	1	10^−3^	0.708	1.000
AREA	Linear	31.622	1	10^−3^	0.625	1.000

Within‐subject effect: N1	*F*		*df*	*p*	η^2^	Power
CONTRAST	4.630		2.577	.006	0.196	0.870
AREA	15.623		3.000	10^−3^	0.451	1.000
AREA*CONTRAST	1.991		7.371	.057	0.095	0.777

Within‐subject contrast	Trend	*F*	*df*	*p*	η^2^	Power
AREA	Linear	41.659	1	10^−3^	0.687	1.000

Within‐subject effect: P2	*F*		*df*	*p*	η^2^	Power
CONTRAST	0.734		3.000	.536	0.037	0.197
AREA	1.212		2.424	.312	0.060	0.274
AREA*CONTRAST	0.757		7.780	.395	0.038	0.131

Neither the total stimulus area undergoing a change in luminance contrast nor the Michelson contrast influenced the latency in the VEP. The highly significant two‐way interaction CONTRAST*COMPONENT and AREA*COMPONENT shows that the influence of these two factors differs between the different VEP components.

C1 latency is unaffected by the total stimulus area undergoing a luminance contrast change. Its latency does respond in a nonlinear manner as Michelson contrast increases. Its latency following offset of the DB25, the dartboard with the least area undergoing a change in luminance contrast, is considerably shorter at the Michelson contrast below the saturation threshold of the magnocellular system than at the contrasts above this threshold. The significant two‐way interaction AREA*CONTRAST indicates that these two factors influence the latency of this component differently. P1 latency decreases linearly as the total stimulus area undergoing a change in luminance contrast increases and decreases linearly as Michelson contrast of this change increases. N1 latency decreases as the total stimulus area undergoing a change in luminance contrast decreases but increases as the Michelson contrast of this change increases. Its latency to the disc at the Michelson contrast below the saturation level of the magnocellular system is clearly shorter than to the disc with a Michelson contrast above this threshold. N2 latency does not respond to either total stimulus area undergoing a luminance contrast change or the Michelson contrast of this change.

## 4 DISCUSSION

The aim of this discussion is to clarify the contribution of the magno‐ and parvocellular systems to the neural response in the course of retinal information processing within the human visual system. We will do so by relating the amplitude and latency of VEP components to the size of the active neural population and its discharge activity to the response properties of these two systems. In line with our previous investigation, we modulated the total stimulus area undergoing a luminance contrast change. This enabled us to attribute neural processing to a mechanism selective to temporal‐ and spatial luminance contrast; stimulus selectivity attributed to the magno‐ and parvocellular systems (Derrington & Lennie, 1984; Robson, 1966).

In addition to the total area undergoing a change in luminance, we also varied the level of the luminance change, that is, the luminance contrast of the elements. This enabled us to relate changes in the VEP to the discharge activity of the active neural population to the contrast response function of the magno‐ and parvocellular systems. Finally, having presented the identical dartboard image as a pattern reversing and pattern onset stimulus, we are able to ascertain the contribution of the phasic and tonic neural response to the VEP and link these to the magno‐ and parvocellular systems.

In this section, we will first discuss with the overall influence of neural population size and its discharge activity on the amplitude of VEP.

### 4.1 Changes in the VEP consistent with response characteristics of the magno‐ and parvocellular systems

The significant two‐way interactions MODE*COMPONENT, AREA*COMPONENT, and CONTRAST*COMPONENT indicate that these factors influenced the neural response at the various stages of processing differently. We will hence examine the influence of varying the size of the active neural population and its discharge activity on individual VEP components for changes reflecting the response characteristics of the magno‐ and parvocellular systems.

The phasic nature of the neural response during temporal luminance contrast processing advocated in our previous work was corroborated by the observation that the P100 and P1 amplitude did not differ when the identical dartboard pattern was viewed as a pattern reversing‐ or pattern onset stimulus.

### 4.2 Changes in amplitude of the first VEP component consistent with characteristic of the magno‐ and parvocellular systems

The N75 amplitude reflects the size of the neural population activated by the temporal luminance contrast property in the stimulus. Selectivity for temporal luminance contrast is associated with the magnocellular system. Its amplitude increases linearly across the luminance contrast level of the dartboard elements, an indication that the discharge level of the active neural population follows the contrast response function of the parvocellular system.

The C1 amplitude following onset of the disc increases linearly with the luminance contrast level of the disc. This indicates that the discharge activity of the neural population processing temporal luminance contrast follows the contrast response function of the parvocellular system.

The C1 amplitude following onset of the three dartboard images exhibits both a linear and nonlinear increase with luminance contrast level of the elements but a decrease with an increase in the size of the neural population responding to the temporal luminance contrast in the image. The two‐way interaction CONTRAST*AREA is consistent with both the temporal‐ and spatial luminance contrast selective mechanism contributing, albeit differently, to the underlying neural response. The former is associated with the magno‐, the latter with the parvocellular system. The presence of a linear and nonlinear increase in amplitude with increasing luminance contrast of the dartboard elements points to the discharge activity that follows both the contrast response function of the magno‐ and parvocellular systems.

The C1 amplitude following the offset of the disc yielded no significant influence of luminance contrast level and did not reach the 1 μV threshold. The C1 amplitude following offset of our dartboard images appears unaffected by either luminance contrast level of their element or by the total stimulus area occupied by these elements. The directed testing revealed a quadratic relationship between the C1 amplitude and the total stimulus area. Its amplitude peaks at the dartboard image with the maximum power in the high spatial frequency spectrum. The change in the (weak) underlying neural response is consistent with a mechanism selective to spatial luminance contrast, that is, the selectivity of the parvocellular system.

### 4.3 Changes in amplitude of the second VEP component consistent with characteristics of the magno‐ and parvocellular systems

The P100 amplitude directly reflects the size of the neural population required to code the temporal luminance contrast property of the image. Its amplitude did not increase linearly with the luminance contrast level of the dartboard elements; a behavior consistent with the discharge activity following the contrast response function of the magnocellular system.

The P1 amplitude following onset of the disc exhibits both a linear and a nonlinear increase with the luminance contrast level of the disc. The jump in its amplitude between the lowest and the next higher luminance contrast level and the small increase in amplitude between the three higher luminance contrast levels is consistent with discharge activity following the contrast response function of the magnocellular system.

When viewing the dartboard images, its amplitude reflects the size of the neural population coding the temporal luminance contrast in the image. However, the P1 amplitude is impervious to the luminance contrast level of the dartboard elements. This indicates that the neural response signals processing of temporal luminance contrast and that the discharge activity of the active neural population is insensitive to the luminance contrast level of the dartboard elements.

The P1 amplitude following offset of the disc matches that observed following its onset. There is a clear discontinuity in its amplitude between the luminance contrast level below saturation threshold of the magnocellular system and the luminance contrast levels above this threshold. This is again consistent with the underlying neural response arising from discharge activity that follows the contrast response function of the magnocellular system.

While the undirected comparison indicated that the P1 amplitude following offset of the dartboard images did not vary with the size of the active neural population, the directed comparison yielded a significant quadratic trend in its amplitude. Interestingly, its amplitude to the DB50 image is now the lowest, rather than the highest as observed following onset of the dartboard images. A similar inversion is also observed with the luminance contrast level. At the lowest luminance contrast level, its amplitude is highest and at the highest luminance contrast level lowest. These findings indicate that the neural response is based on the spatial luminance contrast selective mechanism and that the discharge activity of the neural population involved follows the contrast response function of the parvocellular system and that inhibitory interneurons must modulate the neural response.

### 4.4 Changes in amplitude of the third VEP component consistent with characteristics of the magno‐ and parvocellular systems

The N135 amplitude varied with the active neural population size but not with the discharge level of this population. The directed comparison did reveal a cubic relationship between active neural population size and N135 amplitude. The most parsimonious explanation is that both temporal‐ and spatial luminance contrast mechanisms contribute to the neural response.

The N1 amplitude following onset of the disc only reaches the 1 μV threshold at the two highest luminance contrast level. Lacking a reliable neural response at all luminance contrast levels, we refrain from further discussion of the N1 following disc onset. The N1 amplitude following onset of the dartboard images decreased with the total stimulus area undergoing a luminance contrast change, indicating that the neural response reflects the mechanism selective to spatial luminance contrast. Its markedly lower amplitude at the lowest luminance contrast level and the saturation at the higher luminance contrast levels, something that is particularly evident with the DB25 dartboard, suggest that the neural discharge activity follows the contrast response function of the magnocellular system.

The N1 amplitude following offset of the disc as well as the dartboard images did not reach the 1 μV threshold under most conditions, suggesting that the neural response has subsided.

### 4.5 Changes in amplitude of the fourth VEP component consistent with characteristics of the magno‐ and parvocellular systems

The P240 amplitude is unaffected by the size of the active neural population but increases linearly with the discharge activity of this population. This points to the neural response not being based on a temporal luminance contrast selective mechanism and that the discharge activity of the active neural population follows the contrast response function of the parvocellular system.

As the P2 amplitude following onset of the disc only reaches the 1 μV threshold at the two highest luminance contrast levels, we consider the neural response to have subsided and omit further discussion. The P2 amplitude to the dartboard images reflects the power in the high spatial frequency characteristic of the three dartboard images. This indicates that its underlying neural response reflects spatial luminance contrast processing. The jump in amplitude between lowest and second‐lowest luminance contrast level and the nonlinear relationship between amplitude and luminance contrast level indicates that the discharge activity of the active neural population follows the contrast response function of the magnocellular system.

The P2 amplitude following offset of the disc as well as the dartboard images did not reach the 1 μV to most stimuli, indicating that the neural response has subsided below detectability.

The effects of varying the total stimulus area signaling a change in luminance contrast and the luminance contrast level used on the individual VEP components indicates that the discharge activity during temporal luminance contrast processing follows the contrast response function of the parvocellular system and during spatial luminance contrast processing the discharge activity follows the contrast response function of the magnocellular system.

### 4.6 Changes in VEP latencies consistent with characteristics of the magno‐ and parvocellular systems

Oscillations in the VEP arise from modulation of the ongoing neural response by feedback projections (Foxe & Simpson, 2002; von Stein, Chiang, & Konig, 2000; von Stein & Sarnthein, 2000). Neural processing of the low spatial frequency properties of a stimulus is characterized by high temporal oscillations in the VEP and vice versa (Frund, Busch, Korner, Schadow, & Herrmann, 2007). Magnocellular neurons have a faster conducting axons than their parvocellular counterpart neurons (Schiller & Malpeli, 1978) so that their signal arrives at striate cortex 20 ms ahead of the parvocellular signal (Klistorner, Crewther, & Crewther, 1997; Laycock, Crewther, & Crewther, 2007). Activation latencies of visual areas along the dorsal pathway are also shorter than those along the ventral pathway, implying that axonal conduction velocity between areas of the former is faster than between areas of the latter (Chen et al., 2007). The VEP can be modeled by threating the electric potential at the scalp resulting from the neural response associated with each VEP component as a Gauss function (Marcar & Jäncke, 2016). Interactions between neural processes will hence not only influence the amplitude of the measured potential at the scalp, but also the latency of the individual VEP components.

Both mode of presentation and discharge level of the active neural population had a significant influence on the latencies of the VEP components, while the size of the active neural population did not. The significant two‐way interaction MODE*COMPONENT indicates that the four VEP components were affected differently when viewing the identical dartboard pattern as a pattern reversing or pattern onset stimulus, thus altering the appearance of the VEP and that. This corroborates the findings of our previous investigation. The significant two‐way interactions AREA*COMPONENT and CONTRAST*COMPONENT indicate that changing the total stimulus area undergoing a change in luminance and the level of the luminance contrast influenced the latency of the four VEP components differently. This is consistent with multiple mechanisms processing the retinal input. The significant three‐way interaction AREA*CONTRAST*COMPONENT indicates that the neural response associated with each mechanism interact differently at the various stages of processing the retinal input. In the succeeding sections, we examine the effect of varying the total stimulus area undergoing a change in luminance contrast and the luminance contrast level on the latency of each VEP component for changes that match known response characteristics of the magno‐ and parvocellular systems.

### 4.7 Changes in latency of the first VEP component consistent with characteristics of the magno‐ and parvocellular systems

N75 latency was unaffected by a change in total area undergoing an increase in luminance contrast but strongly affected by a change in luminance contrast level. It was shortest at the lowest luminance contrast level but varied little across the higher luminance contrast levels. This matches the contrast response function of the magnocellular system, where the discharge level saturates above a 16%–32% change in luminance contrast.

C1 latency following onset of the stimulus was unaffected by the change in luminance contrast level in our stimuli but increased linearly with the size of the neural population responding to the temporal luminance contrast. The significant two‐way interaction CONTRAST*AREA is most likely attributable to the difference in neural response during spatial luminance contrast processing of the disc and the dartboard images.

C1 latency following offset of the stimulus increases linearly with as the size of the neural population responding to the temporal change in luminance decreases but is unaffected by the luminance contrast level used. The significant two‐way interaction CONTRAST*AREA indicates that these two factors influence its latency differently. We see this difference as reflecting the influence of the neural response associated with the processing of the spatial luminance contrast content of the disc and the dartboard images.

### 4.8 Changes in latency of the second VEP component consistent with characteristics of the magno‐ and parvocellular systems

P100 latency is unaffected by the size of the neural population processing temporal change in luminance contrast but exhibits a linear and nonlinear behavior across the luminance contrast levels. At the lowest luminance contrast level, P100 latency is markedly longer than at the higher luminance contrast levels. Across the higher luminance contrast levels, its latency actually varies little. An increase in luminance contrast level has P100 latency decrease but that of the N75 increase. We interpret this to indicate that the latency of these two components arises from the interaction of the sink and source electric potential generated during processing of temporal luminance contrast.

P1 latency following onset of the stimulus is unaffected by the luminance contrast level but exhibits both a linear and nonlinear response to an increase with the size of the neural population processing temporal luminance contrast. We take the presence of a nonlinearity to indicate that its latency is also influenced by an interaction of the neural responses arising during temporal‐ and spatial luminance contrast processing.

P1 latency following offset of the stimulus decreased linearly as the size of the neural population processing temporal luminance contrast increased but decreased as the luminance contrast level of the stimuli increased. Because the two‐way interaction CONTAST*AREA is not significant, the influence of these two factors on its latency must be considered comparable.

### 4.9 Changes in latency of the third VEP component consistent with characteristics of the magno‐ and parvocellular systems

N135 latency is unaffected by the size of the neural population processing temporal luminance contrast but increases linearly with the luminance contrast level. Its latency at the lowest luminance contrast level was distinctly longer than at the three higher luminance contrast levels and did not vary across the higher luminance contrast levels. This indicates that to the discharge activity of the active neural population follows the contrast response function of the magnocellular system.

N1 latency following onset of the stimulus is modulated by both the size of the active neural population and the discharge level of this population. The linear relationship between latency and luminance contrast level indicates that the discharge activity of the active neural population follows the contrast response function of the parvocellular system. The nonlinear relationship between latency and the active neural population size indicates that the neural response is connected to the spatial luminance contrast property of the stimulus, that is, the stimulus selectivity associated with the parvocellular system.

N1 latency following offset of the stimulus is modulated by both the size of the active neural population and the discharge activity of this population. The linear relationship between latency and active population size indicates that the latter reflects the temporal luminance contrast property of the image, the stimulus selectivity associated with the magnocellular system.

### 4.10 Changes in latency of the fourth VEP component consistent with characteristics of the magno‐ and parvocellular systems

P240 latency is unaltered by change in the size of the active neural population but decreases linearly with increasing luminance contrast level. The linear relationship between luminance contrast level and its amplitude hides a noteworthy change in its response to luminance contrast level between the DB50 and DB12.5 dartboard images. Recall that the DB50 image has the maximum and the DB12.5 dartboard the minimum high spatial content. When viewing the DB50 dartboard, changing from the lowest to the next highest luminance contrast level has no effect on P240 latency. Viewing the same dartboard at higher luminance contrast levels is accompanied by a stepwise reduction in its latency. When viewing the BD12.5 dartboard, changing from the lowest to the next higher luminance contrast is accompanied by a marked reduction in its latency. Viewing the same dartboard image at higher luminance contrast level does not alter its latency. This observation suggests that the latency of this VEP component reflects the contrast response function of the parvocellular system when viewing the DB50 dartboard image, but reflects the contrast response function of the neural magnocellular system. Why this is the case in unclear, as the neural response during temporal‐ and spatial luminance contrast processing will be stronger when viewing the DB50 than when viewing the DB12.5 dartboard image. This is a matter that will have to be addressed in a future investigation.

P2 latency following stimulus onset is unaffected by the size of the active neural population but exhibits both a linear and nonlinear relationship with luminance contrast level. There is a noteworthy difference in its response to luminance contrast level between the disc and the dartboard images. Changing the luminance contrast level of the disc has no discernible influence on its latency. Changing the luminance contrast of the dartboards results in a marked reduction in latency between the lowest and next higher luminance contrast level but little change in latency across the three higher luminance contrast levels. This difference between the disc and the dartboard images most likely accounts for the nonlinearity in the observed relationship between luminance contrast and P2 latency. The marked difference in latency between lowest and next higher luminance contrast points to the involvement of a neural mechanism signaling the contrast response function of the magnocellular system.

P2 latency following stimulus offset did not react to a change in the size of the active neural population nor to a change in luminance contrast level.

## 5 CONCLUSION

The influence of varying the size of the active neural population on the amplitude of the different VEP components confirmed the presence of two independent processing stages. The initial stage processes temporal luminance contrast, the subsequent stage processes spatial luminance contrast. By setting one luminance contrast below the saturation threshold of the magnocellular system and the remaining above found that the discharge activity during temporal luminance contrast processing follows the contrast response functions of the parvocellular system, while discharge activity during spatial luminance contrast processing reflects the contrast response function of the magnocellular system.

In spite of the complex nature of the interplay between the two systems, careful modulation of stimulus property reveals the involvement of the magno‐ and parvocellular systems at different stages of processing by considering the influence of active neural population size and its discharge activity on the VEP. From the influence of changing the size of the active neural population and its discharge activity on the latency of the different VEP components, it is possible to identify when the neural response generated by the two processing mechanisms interact.
